# Supplementary material for: Transcription Factor TeMADS6 Coregulates Carotenoid Biosynthesis and Chlorophyll Degradation Resulting in Yellow-Green Petal Color of Marigold (Tagetes erecta)
Source: Plants (Basel). 2025 Dec 10;14(24):3763. doi: 10.3390/plants14243763 (PMC12736798; doi:10.3390/plants14243763)
Supplement: Supplementary file 1 [file plants-14-03763-s001.zip › supplementary Figures.pptx]

## Slide 1
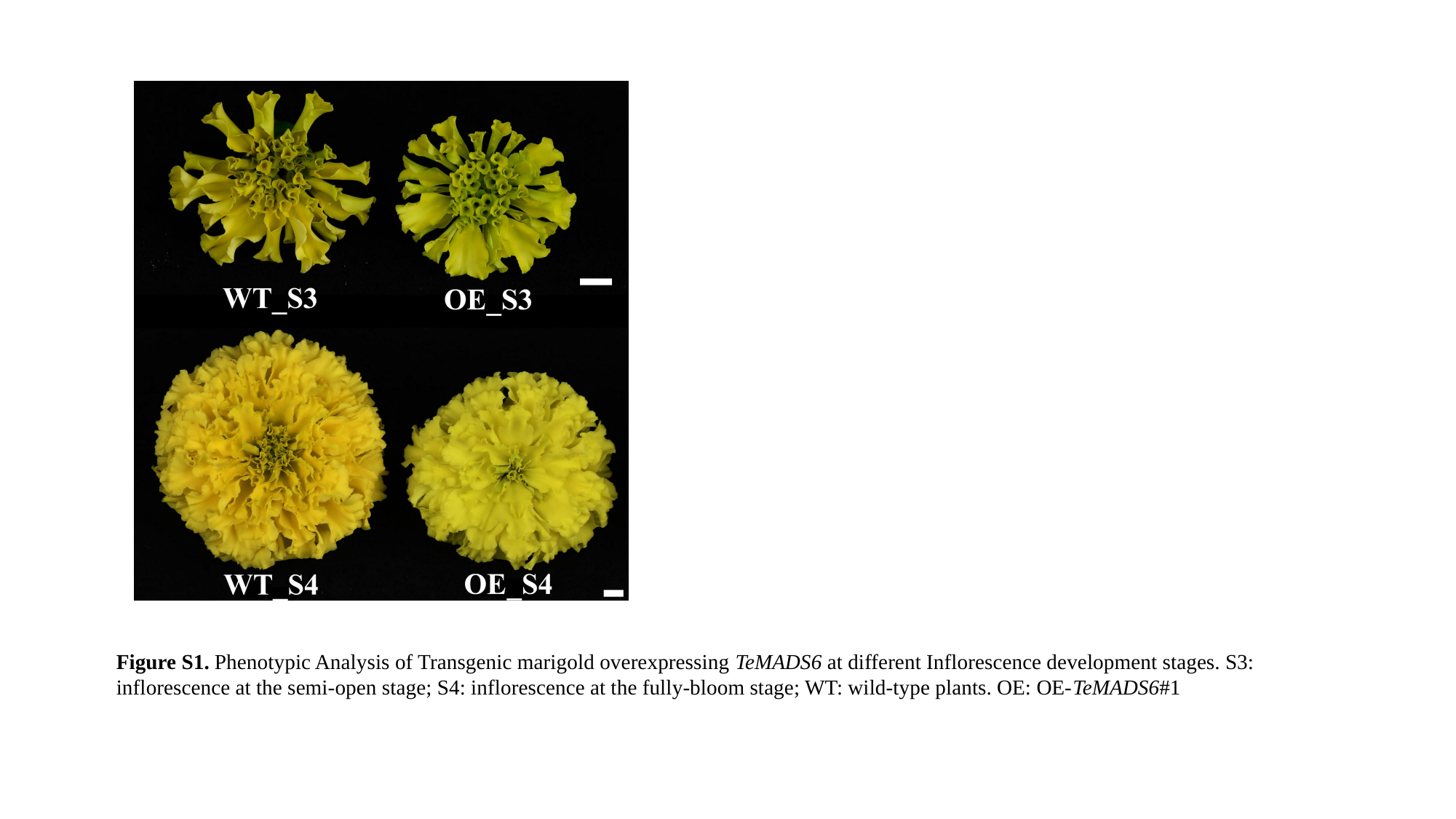

Figure S1. Phenotypic Analysis of Transgenic marigold overexpressing TeMADS6 at different Inflorescence development stages. S3: inflorescence at the semi-open stage; S4: inflorescence at the fully-bloom stage; WT: wild-type plants. OE: OE-TeMADS6#1

## Slide 2
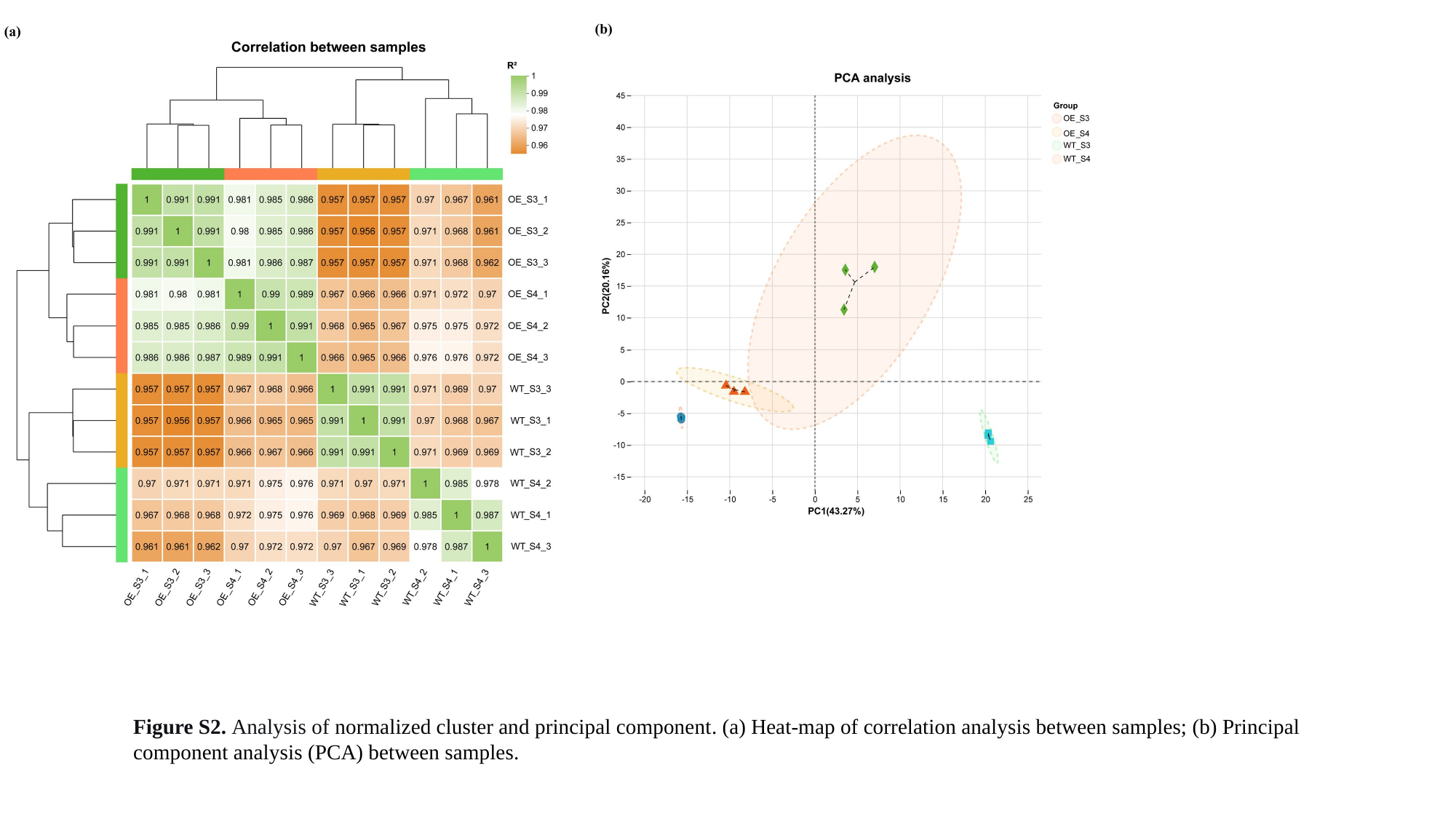

Figure S2. Analysis of normalized cluster and principal component. (a) Heat-map of correlation analysis between samples; (b) Principal component analysis (PCA) between samples.
