# Supplementary material for: Transcription Factor TeMADS6 Coregulates Carotenoid Biosynthesis and Chlorophyll Degradation Resulting in Yellow-Green Petal Color of Marigold (Tagetes erecta)
Source: Plants (Basel). 2025 Dec 10;14(24):3763. doi: 10.3390/plants14243763 (PMC12736798; doi:10.3390/plants14243763)
Supplement: Supplementary file 1 [file plants-14-03763-s001.zip › supplementary Tables.pdf]

Table S1. Sequences of primers.

Primer	Sequence (5′-3′)
RT-TeMADS6-F	AGTGGCGTCTTTTATCGGAGCA
RT-TeMADS6-R	TGAGGCTGCCCATCTGGAATAA
Full-length-TeMADS6-F	AGAAATGGGAAGGGGAAAAGTAG
Full-length-TeMADS6-R	GCATATGACAATCAACTGCTGGG
P2300-TeMADS6-F	gagctcggtacccgg**ggatcc** ATGGGAAGGGGAAAAGTAGAGC
P2300-TeMADS6-R	cttgcatgcctgcag**gtcgac**TCAACTGCTGGGAAAACTAAGTTG
35S-F	ACGCACAATCCCACTATCCTTC
35S-R	TGCTCAACACATGAGCGAAAC
RT-PPH-F	CGTGGACTTACTTGGGTTTGGG
RT-PPH-R	TCCACCAAGACTATTACCGACCA
RT-TeSGR1-F	GAAGTCGTGGCAGAATGGAGAAA
RT-TeSGR1-R	AAGACGAGCAAACAAGTCGAGAA
RT-TeSGR2-F1	ACTTCACCGCCAACCTCACTTT
RT-TeSGR2-R1	TTACTTGAGCCCATTGAGCCAC
RT-TeSGR1-F2	AGTGGCTCAATGGGCTCAAGTA
RT-TeSGR1-R2	TCAAGAACCAACGGCAACTCCT
RT-TeCLH1-F	ATAGCAAAGGAGGGAAAGCAGC
RT-TeCLH1-R	CAATTAGGGCTATAGGCATGGGT
RT-TeNYC1-F	TCAAGCACACCTTTAACAGCCGT
RT-TeNYC1-R	TGCTTGTTCTGAATAGTAGACCCAC
RT-TeNYC2-F	TGGGTTCAGTTTTTCAGGTGGAG
RT-TeNYC2-R	ATTCCTCGGACCAGCTCGACTAA
RT-TeNYC3-F	GATGAGCACTGGAGTGATTTTGT
RT-TeNYC3-R	AATGCCACCAACCCAACTATACC
WSJ=RT-ACT-F	GGGAAATGAATGCCAAAGCCAAG
WSJ=RT-ACT-R	AAGACTTCACAACCACTCTCCAACT
RT-TePSY1-F	AGTGGTTTCAAGGGTGGTGGCTAA
RT-TePSY1-R	GTTTCGCCTCATTATCTTCTTTCG
RT-TePSY4-F	CAACGAGGCTGAAGAAGGTGT
RT-TePSY4-R	AGCATCAACGCAGACCAAACC
RT-TeHYDB-F	GGGGTTCCTTATGGCTTGTTCTT
RT-TeHYDB-R	TCTTCCGTTCCTCCCACTTCT
RT-TeCCD1-2-F	TGGGACAGGAAACACGGCTCT
RT-TeCCD1-2-R	GCAGGTCACCATCCTCCAATAC
RT-TeCCD4b-F	ACGGTCCAGTCCCTCCTTTCCTAA
RT-TeCCD4b-R	GTATTGAGAAAATCGGGACGTCGG
RT-TeCCD7-F	CGACGATGAACATTGATGAAGGA
RT-TeCCD7-R	TTTCACAATTCCCAGTCGCATC
RT-TeNCED1-F	CGGAAATGATTAGCGGTGGA
RT-TeNCED1-R	GGAACTTCAACCCATTTAATACCCG

Note: Lowercase letters are the homologous recombination linker sequence, bold letters are the sequences of specific restriction enzyme cut sites.

Table S2. The information of proteins used in the phylogenetic tree.

Accession numbers	Species	Gene name
AAF19164.1	*Petunia x hybrida*	*PhFBP26*
AAP83396.1	*Petunia x hybrida*	*PhFUL*
AAT07448.1	*Vitis vinifera*	*VvFUL*
NP_001234173.2	*Solanum lycopersicum*	*SlFUL1*
NP_001294867.1	*Solanum lycopersicum*	*SlFUL2*
ACR19996.1	*Vaccinium myrtillus*	*VmTDR4*
QEH04677.1	*Citrus sinensis*	*MADS5*
Q38876.1	*Arabidopsis thaliana*	*AtFULL*
TeORChr2g0068971	*Tagetes erecta*	*TeMADS5*
XM_022128169.2	*Helianthus annuus*	*HaFUL*
BAH36884.1 1	*Chrysanthemum × morifolium*	*CmFUL*
Q38742	*Antirrhinum majus*	*AmSQUA*
lQOJ53903.1	*Tagetes erecta*	*TeSEP3*

Table S3. Sequencing data statistics.

Sample	Q20(%)	Q30(%)	GC content (%)
WT_S3_1	98.94	96.49	42.57
WT_S3_2	98.98	96.6	42.72
WT_S3_3	98.95	96.53	42.79
WT_S4_1	98.99	96.62	42.26
WT_S4_2	98.93	96.44	42.53
WT_S4_3	98.97	96.59	42.51
OE_S3_1	99	96.66	42.76
OE_S3_2	98.96	96.56	42.81
OE_S3_3	98.98	96.58	42.8
OE_S4_1	99.03	96.76	42.7
OE_S4_2	98.98	96.61	43
OE_S4_3	98.93	96.47	42.73

Note: The average value of the clean reads Q20 and Q30 were 98.97% and 96.58%, respectively, and the average GC content was 42.68%.

Table S4. The clean reads were mapped to the reference genome

Sample	Total reads	Total mapped
WT_S3_1	44166010	40832441(92.45%)
WT_S3_2	47743744	44230156(92.64%)
WT_S3_3	46224188	42895493(92.8%)
WT_S4_1	42045208	38620850(91.86%)
WT_S4_2	41164184	37950759(92.19%)
WT_S4_3	47823348	44131867(92.28%)
OE_S3_1	46149370	42421174(91.92%)

OE_S3_2	42713430	39223630(91.83%)
OE_S3_3	43593940	40069258(91.91%)
OE_S4_1	47034772	43405114(92.28%)
OE_S4_2	47007802	43480458(92.5%)
OE_S4_3	42926020	39518750(92.06%)

Note: the total mapping ratio of each library ranged from 91.06% to 92.91% with a perfect average mapping ratio of 92.20%.

Table S5. Transcript length distribution.

Length	Number	Percentage
0~200	2084	3.49%
201~400	4444	7.44%
401~600	4453	7.46%
601~800	4577	7.67%
801~1000	4833	8.10%
1001~1200	4994	8.37%
1201~1400	5168	8.66%
1401~1600	4887	8.19%
1601~1800	4352	7.29%
>1800	19903	33.34%
total	59695	

Note: a total of 59,695 transcripts, among which 33.34% had a length exceeding 1,800 bp.

Table S6. Analysis of annotated unigenes.

	Expre_Gene number	Percent
GO	23653	83%
KEGG	11867	42%
EggNOG	25533	90%
NR	27639	97%
Swiss-Prot	22633	80%
Pfam	23658	83%
Total_anno	27672	97%
Total	28470	100%

Table S7. The transcripts of structure genes related to carotenoid biosynthesis.

Gene ID	WT_S3	OE_S3	Significant (OE_S3_vs_WT_S3)	WT_S4	OE_S4	Significant (OE_S4_vs_WT_S4)	Gene name
TeORChr10g0303291	469.72	95.22	Yes/down	333.33	80.62	Yes/down	*TePSY1*
TeORChr6g019	22.3	18.9	No	19.52	20.1	No	*TePSY*

7071		8			2		2
TeORChr5g0169061	238.15	129.13	No	114.03	155.7	No	*TePSY3*
TeORChr6g0197571	216.94	140.84	No	172.24	150	No	*TePDS*
TeORChr9g0279581	82.7	62.83	No	58.01	81.22	No	*TeCrtISO*
TeORChr10g0299211	92.12	56.3	No	62.2	57.79	No	*TeZISO*
TeORChr10g0302351	280.81	163.1	No	235.26	185.8	No	*TeZDS*
TeORChr1g0030071	160.12	108.6	No	115.29	115.2	No	*TeLCYE*
TeORChr4g0137941	119.19	91.72	No	90.35	99.89	No	*TeLCYB*
TeORChr1g0064991	422.64	175.67	Yes/down	333.36	217.8	No	*TeHYDB*
TeORChr1g0048211	54.04	36.49	No	45.73	37.14	No	*TeHYDE*
TeORChr1g0042531	52.66	52.39	No	34.67	61.7	No	*TeZEP*
TeORChr1g0048621	21.35	44.9	No	39.79	28.54	No	*TeNCED1*
TeORChr9g0287931	134.59	135.46	No	111.23	152.6	No	*TeCCD1*

Table S8. The information of DEGs related to carotenoid biosynthesis.

Group	*TePSY1*	*TeHYDB*
Log_2Fold (OE_S3_vs_WT_S3)/Padjust	-2.19/0	-1.27/6.14e-196
Log_2Fold (OE_S4_vs_WT_S4)/Padjust	-1.97/7.50E-85	/

Note: The slash indicates no significant difference in gene expression levels between the transgenic line and the wild type plants.

Table S9. The transcripts of structure genes related to chlorophyll metabolism.

Gene ID	WT_S3	OE_S3	Significant (OE_S3_vs_WT_S3)	WT_S4	OE_S4	Significant (OE_S4_vs_WT_S4)	Gene Name
TeORChr1g0002951	57.59	35.95	No	46.32	43.63	No	*TeCS*
TeORChr10g0306221	37.87	28.72	No	27.38	31.15	No	*TeRCCR*
TeORChr11g03	136.0	120.	No	102.4	120.	No	*TePPH*

35011	5	96		27	4		*1*
TeORChr6g0203641	32.5	15.027	Yes/down	11.21	15.72	No	*TePPH2*
TeORChr11g0320281	6.98	18.39	No	10.07	21.3	No	*TeSGR1*
TeORChr11g0317241	115.79	96.82	No	49.47	103.3	Yes/up	*TeSGR2*
TeORChr1g0039371	530.95	416.76	No	283.98	405.1	No	*TeSGR3*
TeORChr2g0068541	82.76	49.11	No	68.9	57.47	No	*TeCHLM*
TeORChr9g0276721	167.34	94.53	No	81.2	143.6	No	*TeCHLH*
TeORChr6g0187671	55.43	52.31	No	49.73	56.32	No	*TeCLH*
TeORChr11g0335541	37.03	44.75	No	74.33	69.31	No	*TePORA1*
TeORChr5g0163391	56.98	52.36	No	63.47	53.53	No	*TePORA2*
TeORChr9g0276131	456.33	348.95	No	269.38	398.3	No	*TeCHL27-1*
TeORChr4g0140681	96.58	72.11	No	90.35	79.5	No	*TeCHL27-2*
TeORChr10g0298401	62.7	39.66	No	61.39	40.29	No	*TePAO1*
TeORChr1g0019191	84.01	56.9	No	47.46	73.04	No	*TePAO2*
TeORChr4g0134201	68.57	85.06	No	111.07	87.43	No	*TePAO3*
TeORChr8g0252411	5.07	4.02	No	23.17	5.29	Yes/down	*TeNYC1*

Table S10. The information of DEGs related to chlorophyll metabolism.

Group	*TePPH2*	*TeSGR2*	*TeNYC1*
Log_2Fold (OE_S3_vs_WT_S3)/Padjust	-1.11/1.09E-25	/	/
Log_2Fold (OE_S4_vs_WT_S4)/Padjust	/	1.02/1.67E-05	-2.17/0.006

Note: The slash indicates no significant difference in gene expression levels between the transgenic line and the wild type plants.

Table S11. Statistics of differentially expressed TFs

Comparison	Up (Number)	Down (Number)	Total (Number)
OE_S3_vs_WT_S3	285	65	350

OE_S4_vs_WT_S4	76	64	140

Table S12. Statistics of differentially expressed TF families.

OE_S3_vs_WT_S3		OE_S4_vs_WT_S4	
TF family	Gene number	TF family	Gene number
ERF	64	bHLH	17
NAC	34	NAC	14
MYB	33	HB	13
WRKY	32	MYB	12
bHLH	26	WRKY	12
HB	22	ERF	11
MYB_related	19	MYB_related	11
GRAS	17	Dof	6
GATA	11	bZIP	6
LBD (AS2/LOB)	10	DBB	5
bZIP	10	GATA	5
Dof	9	LBD (AS2/LOB)	5
HSF	9	CO-like	4
MIKC	8	SBP	4
M_type	8	GRAS	3
B3	6	M_type	3
SBP	6	AP2	2
TCP	5	B3	2
ARF	4	BES1	2
YABBY	4	HSF	2
AP2	3	MIKC	4
C3H	3	TCP	2
CO-like	3	ZF-HD	2
DBB	3	ARF	1
HD-ZIP	3	BBR-BPC	1
Nin-like	3	FAR1	1
TALE	3	HD-ZIP	1
BBR-BPC	2	LFY	1
BES1	2	LSD	1
C2H2	2	Nin-like	1
GRF	2	TALE	1
SRS	2	YABBY	1
ZF-HD	2	/	/
FAR1	1	/	/
GeBP	1	/	/
LFY	1	/	/
LSD	1	/	/
NF-YA	1	/	/

RAV	1	/	/
